# Significant Differences in Intestinal Bacterial Communities of Sympatric Bean Goose, Hooded Crane, and Domestic Goose

**DOI:** 10.3390/ani14111688

**Published:** 2024-06-05

**Authors:** Jing Yin, Dandan Yuan, Ziqiu Xu, Yuannuo Wu, Zhong Chen, Xingjia Xiang

**Affiliations:** 1 School of Resources and Environmental Engineering, Anhui University, Hefei 230601, China; yinjing01102023@163.com (J.Y.); yuandandan415@163.com (D.Y.); 13856169756@163.com (Z.X.); wuyuannuo2022@163.com (Y.W.); 2 Anhui Shengjin Lake Wetland Ecology National Long-Term Scientific Research Base, Chizhou 247230, China; 3 International Collaborative Research Center for Huangshan Biodiversity and Tibetan Macaque Behavioral Ecology, Hefei 230601, China

**Keywords:** bean goose, hooded crane, domestic goose, gut bacterial community, pathogens

## Abstract

**Simple Summary:**

The gut microbiota plays important roles for maintaining the health of the host. In this study, the results revealed significant differences in the gut bacterial communities among bean geese, hooded cranes, and domestic geese. In comparison to domestic geese, the gut bacterial community of bean geese and hooded cranes had a greater capacity for energy metabolism, suggesting that wild birds may rely more on their gut microbiota to survive in cold conditions. Furthermore, pathogens were discovered to overlap among the three hosts, reminding us to monitor the potential for pathogen transmission between poultry and wild birds. Hooded cranes showed the highest diversity and relative abundance of pathogens compared to the other two species. Due to its vulnerable status, more focus should be paid to the protection of the hooded crane species. These findings could help us gain a deeper understanding of the structure of gut bacterial and pathogenic communities in poultry and wild birds.

**Abstract:**

The host’s physiological well-being is intricately associated with the gut microbiota. However, previous studies regarding the intestinal microbiota have focused on domesticated or captive birds. This study used high-throughput sequencing technology to identify the gut bacterial communities of sympatric bean geese, hooded cranes, and domestic geese. The results indicated that the gut bacterial diversity in domestic geese and hooded cranes showed considerably higher diversity than bean geese. The gut bacterial community compositions varied significantly among the three hosts (*p* < 0.05). Compared to the hooded crane, the bean goose and domestic goose were more similar in their genotype and evolutionary history, with less difference in the bacterial community composition and assembly processes between the two species. Thus, the results might support the crucial role of host genotypes on their gut microbiota. The gut bacteria of wild hooded cranes and bean geese had a greater capacity for energy metabolism compared to domestic geese, suggesting that wild birds may rely more on their gut microbiota to survive in cold conditions. Moreover, the intestines of the three hosts were identified as harboring potential pathogens. The relative abundance of pathogens was higher in the hooded crane compared to the other two species. The hooded crane gut bacterial community assemblage revealed the least deterministic process with the lowest filtering/selection on the gut microbiota, which might have been a reason for the highest number of pathogens result. Compared to the hooded crane, the sympatric bean goose showed the least diversity and relative abundance of pathogens. The intestinal bacterial co-occurrence network showed the highest stability in the bean goose, potentially enhancing host resistance to adverse environments and reducing the susceptibility to pathogen invasion. In this study, the pathogens were also discovered to overlap among the three hosts, reminding us to monitor the potential for pathogen transmission between poultry and wild birds. Overall, the current findings have the potential to enhance the understanding of gut bacterial and pathogenic community structures in poultry and wild birds.

## 1. Introduction

The gut microbiota plays a crucial role in maintaining the well-being of various animal species. In addition to aiding in digestion [1], regulating metabolism, and bolstering immunity [2], microbial communities may also protect against pathogen invasion [3]. Changes in the gut microbiota increase the host’s susceptibility to diseases [4]. Furthermore, multiple factors have the potential to modify the structure and function of the gut microbiome, such as dietary choices [5], environmental conditions [6], gender differences [7], and fluctuations in the seasons [8]. Previous studies have claimed that the host genotype has a great impact on the composition of the gut microbiota [9].

In contrast to other vertebrates, the gut microbiota of migratory birds is markedly different due to their distinctive physiological characteristics [10]. The homeostasis of the intestinal microbiota plays a vital role in the growth and well-being of avian species [11]. It is essential for proper nutrient absorption [12], maintaining a healthy immune system [13], and maintaining different physiological functions [14]. The intestinal microbiota comprises crucial symbionts that profoundly influence the host’s life [15], while the host is also an essential factor influencing the intestinal microbiota [16]. However, migratory birds may be susceptible to disease. Wild birds inhabit various habitats during long-distance migration, which raises the potential for pathogen invasion. With overlapping niches, migratory birds are capable of spreading their intestinal pathogens to other sympatric host species [17,18]. The transmission of these pathogens to poultry, humans, and other wild birds can occur as a result of bird migration [19,20]. Therefore, a better understanding of the potential pathogens present in the intestinal tracts of migratory birds may contribute to the elucidation of cross-species pathogen transmission.

The hooded crane, scientifically known as *Grus monacha*, is a large waterfowl species that undertakes long-distance migrations. It is classified as vulnerable on the International Union for the Conservation of Nature (IUCN) red list of threatened species [21]. Another large wintering migratory wild bird is the bean goose (*Anser fabalis*). The bean goose is one of the most abundant wintering birds in the Yangtze River floodplain. The wintering period of these two migratory birds is from October to April in the Yangtze River floodplain. Anthropic activities trigger the rapid degradation of lake wetlands, leading to a significant reduction in food availability. Shengjin Lake, an internationally important wetland, is a river-connected shallow lake in the middle of the Yangtze River floodplain. During winter, the Shengjin Lake provides suitable foraging habitats for wild migratory birds, making it a significant wetland [22]. More than 12,000 bean geese and ≥350 hooded cranes perish annually in Shengjin Lake [23,24]. Wild birds typically assemble on farmland to rest and forage during winter [25]. However, there is a substantial population of poultry in farmland. The potential for the increased cross-transmission of pathogens exists due to the shared foraging niches of the bean goose, hooded crane, and domestic goose (*Anser anser*). The current study aimed to elucidate the composition of the gut bacterial communities and identify possible pathogens in bean geese, hooded cranes, and domestic geese during the wintering period in Shengjin Lake.

In this study, we used high-throughput sequencing technology to investigate the gut bacterial communities and infer the potential pathogens of sympatric bean geese, hooded cranes, and domestic geese at Shengjin Lake. We propose the following hypotheses: (a) the differences in the gut bacterial communities could be shaped by multiple factors, such as host genotype, diet, and living environment; (b) wild birds (i.e., bean geese and hooded cranes) might carry more diverse potential pathogens due to their harsh living environment relative to domestic geese.

## 2. Materials and Methods

### 2.1. Ethics Statement

Non-invasive fecal sample collection did not involve the hunting of experimental animals. Permission for this study was obtained from the Committee on Laboratory Animal Ethics and Regulation of Anhui University (IACUC-2024-043).

### 2.2. Sample Collection

In this study, the samples were collected via a non-invasive sampling method (fecal sampling techniques). Bean geese, hooded cranes, and domestic geese often forage together in rice paddies [23,25]. The diets of the three hosts are shown in Table S1 [26,27,28,29,30,31]. At the same rice paddies, fecal samples were collected from bean geese and hooded cranes. The area was inhabited by approximately 100 hooded cranes, 400 bean geese, and 60 domestic geese. Fecal samples were collected from bean geese, hooded cranes, and domestic geese on the 15th of November 2018. First, a telescope was used to find areas where bean geese and hooded cranes mixed flocks for foraging. The fresh fecal samples were collected immediately after the completion of the birds’ foraging activity. The fecal samples of domestic geese were then obtained in the yard of a farmer. To reduce the risk of contamination, the outermost layer of each sample was removed. Each fresh fecal sample was collected separately in a 15 mL centrifuge tube (sterilized), with ≥2 g per sample. As a precaution against sample contamination, polyethylene gloves were discarded and replaced after collecting the fecal samples. Moreover, to avoid redundancy, the samples were obtained at 10 m intervals. The feces were temporarily stored in an insulated container with ice packs after collection. They were returned to the laboratory within 12 h and maintained at −20 °C.

### 2.3. Bird Species Determination

The Total DNA was extracted from the fecal samples using the Qiagen DNA Stool Mini Kit (Hilden, Germany). The bird species were identified via the sequencing of the mitochondrial COI gene [32,33]. This section’s specific details are included in the Supplementary Materials. After species determination, a total of 60 samples were used in this study, with 20 samples per species (i.e., bean geese, hooded cranes, and domestic geese).

### 2.4. PCR and Amplicon Library Preparation

The bacterial 16S rRNA gene fragment of the V4-V5 hypervariable regions was amplified before sequencing using the primer sets F515/R907 [34,35]. The Supplementary Materials comprise the details of this section.

### 2.5. Bioinformatics

The raw bacterial data were examined via qiime2-2021.2 software [36]. Low-quality sequences were removed using the “deblur” algorithm [37]. The remaining high-quality sequences were grouped into amplicon sequence variations (ASVs) with 100% similarity. The evaluation of the chimeras was performed using the “vsearch” technique [38]. The annotation process was performed via the “classify-sklearn” algorithm using the Silva database v. 132. The subsets of 15,000 sequences per sample were randomly selected for further analyses.

### 2.6. Statistical Analysis

The normal distribution was examined using Shapiro–Wilk’s test for the gut bacterial data (i.e., the bacterial diversity and relative abundance; Table S2). The non-normal distribution data were transformed using a square root transformation to achieve a normal distribution (Table S2). A one-way analysis of variance (ANOVA) was performed to examine the differences in the gut bacterial data. The three hosts were comparatively analyzed using linear discriminant analysis effect size (LEfSe) and the non-parametric Kruskal–Wallis rank-sum test (*p* ≤ 0.05; LDA threshold ≥ 2) to detect the biomarkers in the gut bacterial taxa [39]. The community composition was analyzed using non-metric multidimensional scaling (NMDS) and a similarity analysis (ANOSIM) (permutations = 999) in R-Studio (V.4.2.1) using a vegan package (V.2.6-4). The identification of the unique and common gut bacterial ASVs among the three hosts was carried out using a Venn diagram (https://bioinfogp.cnb.csic.es/tools/venny/index.html, accessed on 24 May 2024). The labdsv package (V.2.1-0) in R-Studio (V.4.2.1) was used to examine the indicator species of the gut bacteria in each host. The beta nearest taxon index (betaNTI) was calculated using the picante package and Phylocom 4.2 to analyze the gut bacterial community assembly of the three hosts [40]. A PICRUSt analysis was used to predict the gut bacterial function [41,42]. A co-occurrence network analysis was conducted using R-Studio (V.4.2.1) and Gephi v.0.9.2 software [43]. The potential pathogens were identified via the functional annotation of prokaryotic taxa (FAPROTAX) database [44] and searched as keywords in the Web of Science to identify their hosts and symptoms [45,46,47,48,49,50,51,52,53,54,55,56,57,58,59,60,61,62,63,64]. The information of pathogens was shown in Table S3.

## 3. Results

### 3.1. Bacterial Diversity

**Figure 1:**
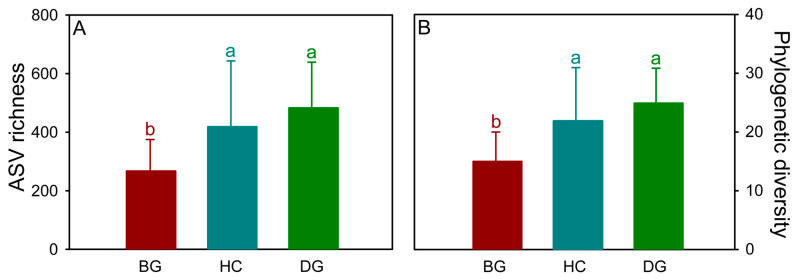
Intestinal bacterial ASV (amplicon sequence variant) richness (**A**) and phylogenetic diversity (**B**) in the three hosts. The letters represent significant differences from the one-way ANOVA (analysis of variance) test (*p* < 0.05; n = 20 for the BG, HC, and DG, respectively). BG: bean goose; HC: hooded crane; DG: domestic goose; ASV: amplicon sequence variation; ANOVA: analysis of variance.

In total, 1,628,677 bacterial sequences passed the quality filter, and 2,466 bacterial ASVs were discovered in all samples. The number of sequences per sample ranged from 15,790 to 50,660, while the number of ASVs per sample ranged from 65 to 714. Among the three species, a total of 545 ASVs, representing 23.7% of the total, were shared in their guts (Figure S1). The number of distinct bacterial ASVs was 155 (6.7%) for the bean goose, 359 (15.6%) for the hooded crane, and 487 (21.2%) for the domestic goose. The phylogenetic diversity and ASV richness were used to evaluate the bacterial alpha diversity. The diversity of the hooded crane and domestic goose was significantly higher than that of the bean goose (Figure 1).

### 3.2. Bacterial Community Composition

The dominant gut bacterial phyla were Firmicutes (49.6%), Proteobacteria (18.8%), Actinobacteria (6.00%), and Bacteroidetes (1.5%). The dominant gut bacterial genera were *Lactobacillus* (32.5%), *Pseudomonas* (6.60%), *Enterococcus* (4.26%), and *Bacillus* (1.49%). The hooded crane showed a greater relative abundance of Actinobacteria and *Bacillus*. In comparison to the other two species, the relative abundance of Proteobacteria and *Pseudomonas* was greater in the domestic goose (Figures S2 and S3). The domestic goose had a substantially lower intestinal Firmicutes/Bacteroidetes ratio (F/B) than the bean goose and hooded crane (Figure S4).

**Figure 2:**
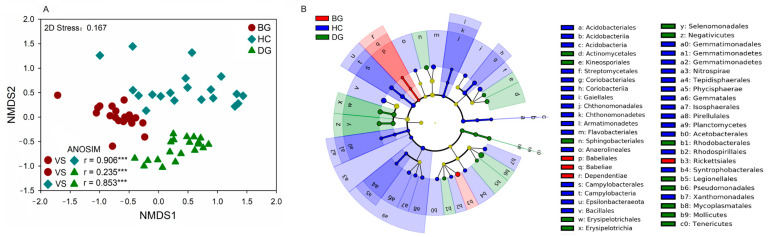
The intestinal bacterial community composition among three hosts (**A**). The LEfSe (linear discriminant analysis effect size) analysis showed intestinal bacterial biomarkers with an effect size > 2, and the alpha value was <0.05 associated with each species (**B**). (***: *p* < 0.001; n = 20 for the BG, HC, and DG, respectively); ANOSIM: analysis of similarity; BG: bean goose; HC: hooded crane; DG: domestic goose.

The community compositions of the intestinal bacteria varied considerably among the three species (Figure 2A). The LEfSe showed that the bacteria from one phylum (Dependentiae), one class (Babeliae), and two orders (Babeliales and Rhckettsiales) were enriched in the gut of the bean goose. The bacteria from six phyla (Nitrospirae, Planctomycetes, Armatimonadetes, etc.), six classes (Coriobacteriia, Chthonomonadetes, Campylobacteria, etc.), and eight orders (Coriobacteriales, Campylobacterales, Bacillales, etc.) were enriched in the gut of the hooded crane. One phylum (Tenericutes), three classes (Mollicutes, Negativicutes, and Erysipelotrichales), and nine orders (Actinomycetales, Kineosporiales, and Tenericutes) were enriched in the domestic goose (Figure 2B and Figure S5).

Bacterial species related to species specificity were identified via an indicator analysis. There were five (e.g., *Streptococcus suis*, *Kaistia geumhonensis*, *Agaricicola taiwanensis,* etc.), ten (e.g., *Paenibacillus xylanilyticus*, *Campylobacter canadensis*, *Allorhizobium vitis,* etc.) and seventeen (e.g., *Lactobacillus aviarius*, *Pseudomonas lundensis*, *Clostridium akagii,* etc.) indicator bacterial species in the guts of the bean goose, hooded crane, and domestic goose, respectively (Table 1).

### 3.3. Gut Bacterial PICRUSt Analysis

**Figure 3:**
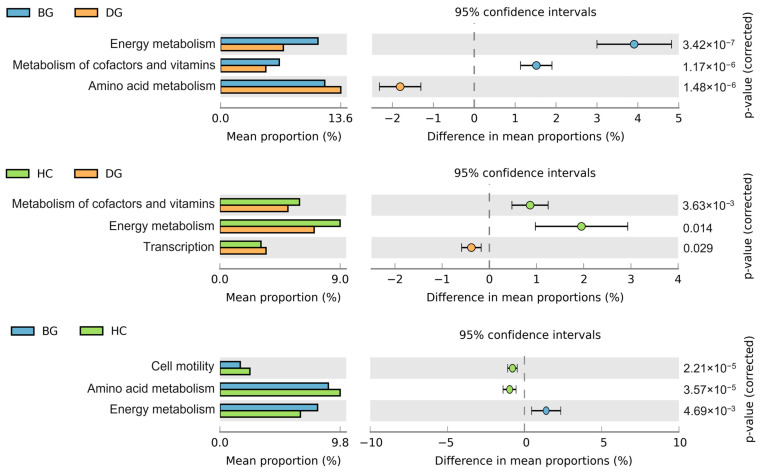
The difference in gut bacterial potential functions predicted using a PICRUSt (phylogenetic investigation of communities by reconstruction of unobserved states) analysis among three different hosts (*p* < 0.05; n = 20 for the BG, HC, and DG, respectively). BG: bean goose; HC: hooded crane; DG: domestic goose.

Based on the KEGG database, gut bacteria have various important roles, such as energy metabolism, cofactor and vitamin metabolism, amino acid metabolism, transcription, and cell motility. Based on the PICRUSt analysis, it was found that the bean goose and hooded crane displayed higher capacities for energy metabolism and the metabolism of cofactors and vitamins compared to the domestic goose (Figure 3). The domestic geese showed improved amino acid metabolism and transcription capacity in comparison to the bean goose and hooded crane. The bean geese had a higher potential for energy metabolism than the hooded crane. The hooded crane showed greater cell motility and amino acid metabolism capacity compared to the bean goose (Figure 3).

### 3.4. Gut Bacterial Community Assembly

**Figure 4:**
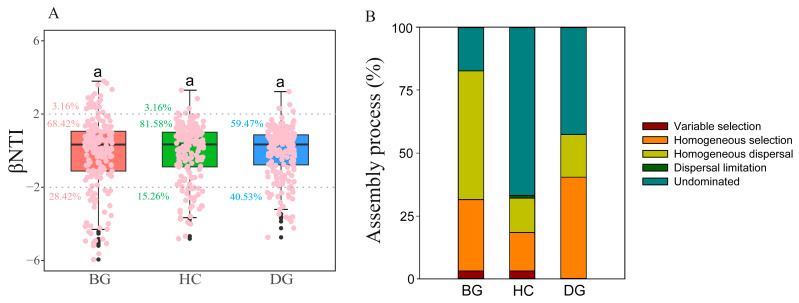
The bacterial community assembly processes in the guts of three species. The values of the weighted beta nearest taxon index (betaNTI) for bacterial communities (**A**). The percentage of deterministic processes (i.e., homogeneous and variable selection) and stochastic processes (i.e., dispersal limitation, homogenizing dispersal, and undominated) in the guts of three species (**B**). The letters represent significant differences from the one-way ANOVA (analysis of variance) test (*p* > 0.05; n = 20 for the BG, HC, and DG, respectively).

The values of the betaNTI (beta nearest taxon index) within the range from −2 to 2 suggest a stochastic process, while values ≥ 2 or ≤−2 indicate deterministic processes. In the guts of bean geese, hooded cranes, and domestic geese, most of the betaNTI scores for the bacterial community assembly dropped within the range from −2 to 2. This indicates that stochastic assembly played a significant role in shaping the dynamics of the gut bacterial communities in these three species (Figure 4A). The assembly of the gut bacterial community in hooded cranes was associated with the highest stochastic assembly (81.58%) and the least deterministic process (18.42%). In the gut of the bean goose, homogeneous dispersal comprised the prevailing stochastic assembly process at 51.05%. Conversely, in the intestines of hooded cranes (66.84%) and domestic geese (42.63%), the undominated stochastic assembly process was predominant (Figure 4B).

### 3.5. Gut Bacterial Network Analysis

The interrelationships between the bacterial taxa in the guts of the bean goose, hooded crane, and domestic goose were investigated via a co-occurrence network analysis. Most of the interactions within the intestinal network were linked to Proteobacteria (Figure S6). In contrast to the domestic goose and hooded crane, the bean goose demonstrated the least average path length and modularity while also showing the maximum clustering coefficient and average degree (Table S4). In comparison to the domestic goose and hooded crane, the bean goose showed the highest level of network stability as assessed using natural connectivity (Figure S7).

### 3.6. Gut Potential Pathogens

**Figure 5:**
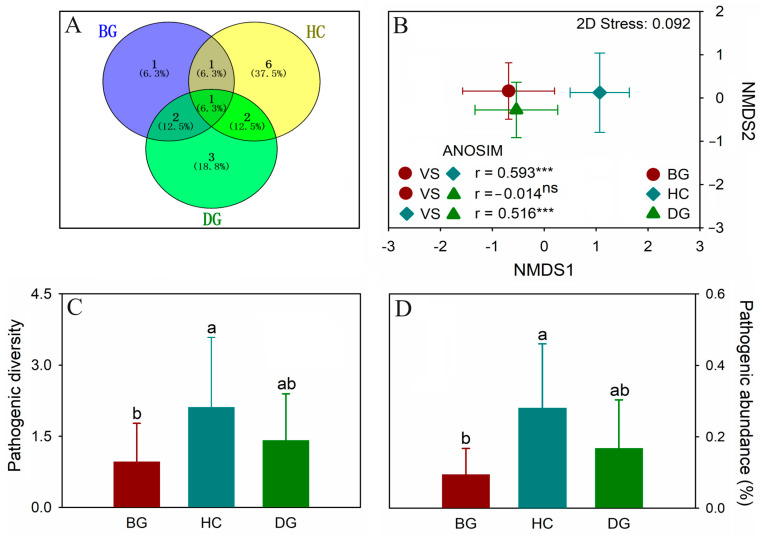
Venn diagram showing the unique and shared animal pathogen ASVs (amplicon sequence variants) in the guts of three hosts (**A**); pathogenic community composition among three species (**B**); animal pathogen diversity (i.e., pathogenic ASVs) (**C**); relative abundance of animal pathogens (**D**). The letters represent significant differences from the one-way ANOVA (*p* < 0.05; n = 20 for the BG, HC, and DG, respectively); ***: *p* < 0.001; ns: no significant difference. BG: bean goose; HC: hooded crane; DG: domestic goose; ASV: amplicon sequence variation.

A total of sixteen potentially pathogenic ASVs were identified, of which 6.3% (one ASV) was shared by three species, 12.5% (two ASVs) by bean geese and domestic geese, 12.5% (two ASVs) by hooded cranes and domestic geese, and 6.3% (one ASV) by bean geese and hooded cranes (Figure 5A). The distinct pathogenic ASVs identified in the guts of the bean goose, hooded crane, and domestic goose were one, six, and three, respectively (Figure 5A). The gut pathogenic community composition of the hooded crane exhibited a significant variation compared to the other species (Figure 5B). It appeared that the gut of the hooded crane had a higher diversity and relative abundance of potential pathogens compared to the other two species (Figure 5C,D).

## 4. Discussion

The focus on avian gut microbiota has been growing, emphasizing the important role of the host species in the formation of the gut microbiota [65,66]. Previous studies have shown that host species with close phylogenetic relationships are responsible for providing the same ecological niche for microbial communities [67,68]. Species with similar genotypes tend to have similar gut microbiota [69]. The bean geese and domestic geese are classified under the same genus, *Anser*, in the coevolutionary branch of the avian phylogenetic tree [70], with less difference in the bacterial community composition and assembly processes between the two species (r = 0.235) relative to the hooded crane (r = 0.906 for the bean goose and hooded crane; r = 0.853 for the domestic goose and hooded crane) (Figure 2A). Thus, the results might support the crucial role of the host phylogenetic relationship (i.e., the genotype) on their gut microbiota [71]. However, different living environments may lead to significant differences in the gut microbiota of closely related species [68]. The living environment of domestic geese is different from that of bean geese, triggering differences in their gut bacterial community composition. In addition, the gut microbiota could be regulated by the host’s diet [72]. The three hosts have different diets during the wintering period (Table S1), which might be another important reason for the differences in the gut bacterial communities among the three species (Figure 1, Figure 2A and Figure 4).

Consistent with the findings from studies on other avian species, such as *Emberiza spodocephala* [73], *Falco tinnuculus* [74], and *Larus relictus* [75], the avian gut microbiota was predominately composed of Firmicutes and Proteobacteria (Figure S2). In contrast to the hooded crane and bean goose, the domestic goose showed a significantly lower ratio of Firmicutes to Bacteroidetes (Figure S4). A larger ratio of Firmicutes to Bacteroidetes contributes to more effective calorie absorption and provides protection against intestinal infections for hosts [76]. The domestic goose displayed abundant *Lactobacillus aviarius* and *Pseudomonas lundensis* (Table 1). Previous studies have shown that these two specific species can inhibit the growth of pathogens in the host [77,78,79,80]. These results suggest that wild birds and domestic poultry might adopt different strategies to prevent pathogen invasion. The present study employed the PICRUSt analysis to predict the functions of the gut bacteria. The results indicated that wild birds have great potential for energy metabolism (Figure 3), which was consistent with the previous findings demonstrating a higher ratio of Firmicutes to Bacteroidetes and efficient calorie absorption in wild birds (Figure S4). Wild birds residing in natural habitats throughout the winter season experience harsh cold conditions, necessitating strong energy metabolism. The living conditions of domestic geese are superior to those of wild birds. Thus, wild birds may rely more extensively on their gut flora to enhance energy metabolism.

Moreover, 10 potential pathogen ASVs that can infect humans and/or animals were identified in three distinct hosts (Table S3). The gut pathogenic community composition showed little difference between the bean goose and domestic goose (Figure 5B). When sampling, mixed flocks with very similar niches were usually found between the two species, resulting in pathogenic transmission and similar pathogenic community composition. The intestinal pathogenic composition of the hooded crane was significantly distinct from that of the other two species (Figure 5B). The hooded crane also showed great diversity and relative abundance of gut animal pathogens (Figure 5C,D). The assembly of the gut bacterial community in hooded cranes was associated with the least deterministic process (Figure 4), leading to a reduced filtration effect on their gut microbiota and resulting in higher pathogens [66]. The hooded crane is classified as an endangered species. Therefore, it is crucial to prioritize the protection of this vulnerable species.

However, the relative abundance and diversity of pathogens were least prevalent in the bean goose compared to the other species. Bean geese appeared healthier than hooded cranes despite inhabiting a comparable niche in a harsh environment during the winter conditions. The population size was found to be more pronounced for the bean goose (≥12,000) compared to the hooded crane (≤350), indicating that bean geese are more suitable for surviving in wintering conditions compared to hooded cranes. Further analyses demonstrated the highest stability along with the greatest complexity in the gut bacterial co-occurrence network of bean geese (Figure S7). A network that has greater stability could potentially contribute to enhanced resilience against unfavorable conditions and facilitate multifunctionality [81]. This, in turn, may assist the bean goose in reducing the risk of pathogen invasion.

During winter, both wild migrating birds and domestic poultry use the same ecological niche for foraging in fields. This study also revealed the presence of a specific proportion of total pathogen ASVs shared in wild birds and poultry (Figure 5A). Previous studies have demonstrated the transmission of diseases between hosts through niche overlaps or close contact [82]. Thus, there might be cross-transmission of pathogens among the bean goose, hooded crane, and domestic goose. Most of the pathogens identified in the guts of the three species have the potential to induce diseases in humans (Table S2). Thus, the possibility of foraging ecological niche overlap may contribute to the transmission of diseases from avian species to poultry, which results in human transmission [18,31]. It is crucial to closely monitor the pathways of pathogenic transmission between wild birds and poultry to accurately assess the potential for disease outbreaks in future studies.

## 5. Conclusions

In conclusion, the results revealed significant differences in the bacterial communities within the guts of the bean goose, hooded crane, and domestic goose. In comparison to domestic geese, wild birds may rely more heavily on their gut microbiota to enhance energy metabolism and adapt to harsh winter conditions. Furthermore, pathogens were detected in the guts of the three hosts, with the hooded crane having the highest diversity and relative abundance of pathogens. Due to its vulnerable status, more focus should be paid to the protection of the hooded crane species. The bean goose displayed less variation and relative prevalence of diseases, indicating that bean geese possess a robust capacity to adapt to their surroundings. These results indicate that it is crucial to prioritize further studies into the transmission of gut pathogens between wild birds and poultry. However, there were certain limitations in this study. Firstly, only three species were included in this study. Secondly, we did not measure the detailed information on the niche overlap degree between different species. These limitations should be clarified in future studies.

## Figures and Tables

**Table 1:** An indicator analysis was conducted to show the indicator species with relative abundances > 0.01% of each host (n = 20 for the BG, HC, and DG, respectively). BG: bean goose; HC: hooded crane; DG: domestic goose.

	Indicator Value	*p*	Taxonomy	Relative Abundance (%)
BG	0.386	0.022	s_*Streptococcus suis*	0.260
	0.469	0.001	s_*Kaistia geumhonensis*	0.112
	0.549	0.001	s_*Agaricicola taiwanensis*	0.068
	0.599	0.001	s_*Methylobacterium hispanicum*	0.058
	0.250	0.008	s_*Clostridium colinum*	0.010
HC	0.905	0.001	s_*Paenibacillus xylanilyticus*	1.806
	0.608	0.001	s_*Campylobacter canadensis*	0.896
	0.638	0.001	s_*Allorhizobium vitis*	0.231
	0.483	0.007	s_*Novosphingobium barchaimii*	0.086
	0.404	0.024	s_*Xanthomonas sacchari*	0.082
	0.426	0.031	s_*Clostridium gasigenes*	0.061
	0.317	0.005	s_*Stenotrophomonas rhizophila*	0.044
	0.300	0.005	s_*Anaerobiospirillum succiniciproducens*	0.038
	0.401	0.001	s_*Mesorhizobium ciceri*	0.031
	0.300	0.003	s_*Campylobacter jejuni*	0.012
DG	0.539	0.003	s_*Lactobacillus aviarius*	78.87
	0.732	0.001	s_*Pseudomonas lundensis*	11.46
	0.597	0.001	s_*Clostridium akagii*	0.923
	0.919	0.001	s_*Clostridium vincentii*	0.561
	0.832	0.001	s_*Sanguibacter inulinus*	0.523
	0.282	0.044	s_*Sphingobacterium faecium*	0.315
	0.299	0.006	s_*Chryseobacterium balustinum*	0.312
	0.365	0.004	s_*Pseudomonas graminis*	0.161
	0.250	0.007	s_*Lactobacillus siliginis*	0.092
	0.200	0.030	s_*Clostridium acidisoli*	0.056
	0.230	0.025	s_*Sphingomonas faeni*	0.043
	0.423	0.008	s_*Rhodococcus hoagii*	0.038
	0.400	0.002	s_*Weissella soli*	0.030
	0.237	0.047	s_*Pseudokineococcus lusitanus*	0.015
	0.299	0.004	s_*Clostridium bovipellis*	0.011
	0.350	0.001	s_*Massiliomicrobiota timonensis*	0.011
	0.225	0.030	s_*Paenibacillus turicensis*	0.010

## Data Availability

The raw data were submitted to the Sequence Read Archive (SRA) of NCBI under the accession number SAMN38055501-SAMN38055502-SAMN38055503.

## Supplementary Materials

The following supporting information can be downloaded at: https://www.mdpi.com/article/10.3390/ani14111688/s1, Table S1: The different diets of three hosts, Table S2: The distribution of data was analyzed by Shapiro-Wilk’s test. Table S3: The potential pathogens were detected in guts of bean goose, hooded crane and domestic goose, Table S4: Co-occurrence network topological features, Figure S1: The Venn diagram showing the unique and shared gut bacterial ASVs in guts of bean goose, hooded crane and domestic goose, Figure S2: Relative abundance of the dominant bacterial phyla, Figure S3: Relative abundance of the dominant bacterial genus, Figure S4: The ratio of relative abundance of Firmicutes and Bacteroidetes, Figure S5: Identified phylotype biomarkers ranked by effect size with the alpha value was <0.05 in different hosts. The phylotype biomarkers were identified as significantly abundant when samples in bean goose, hooded crane and domestic goose were compared, Figure S6: The co-occurrence network structure of gut bacterial community for bean goose, hooded crane and domestic goose. Figure S7: The stability of co-occurrence network. References [20,26,27,28,29,30,31,32,33,34,35,45,46,47,48,49,50,51,52,53,54,55,56,57,58,59,60,61,62,63,64] are cited in the Supplementary Materials.
