# Supplementary material for: Significant Differences in Intestinal Bacterial Communities of Sympatric Bean Goose, Hooded Crane, and Domestic Goose
Source: Animals (Basel). 2024 Jun 5;14(11):1688. doi: 10.3390/ani14111688 (PMC11170997; doi:10.3390/ani14111688)
Supplement: Supplementary file 1 [file animals-14-01688-s001.zip › animals-3023497-supplementary.pdf]

Supporting Information

Supporting Materials and Methods

Bird species determination

The mitochondrion COI barcode area was amplified to confirm whether the DNA samples belonged to the bean geese or hooded cranes [32]. PCR reaction was carried out in 50 μl reaction mixtures, following these parameters: 95℃ for 5 min; 35 cycles of 95°C for 30 s, 55°C for 45 s, and 72°C for 90 s, with a final extension period of 10 min at 72°C. The PCR products were sequenced and blasted (> 99% identity) in National Center for Biotechnology Information (NCBI) to identify bird species [33]. Bean geese and hooded cranes samples were retained for High-throughput sequencing.

PCR and amplicon library preparation

Each sample's 50 ng pure DNA aliquot was used as an amplification template. The V4-V5 hypervariable portions of the bacterial 16S rRNA gene fragments were amplified using primer sets F515/R907 [34], which were coupled with unique identifier tags and sequencing adapters. The amplification was performed on the Illumina Mi-Seq platform (PE 300) at Majorbio in Shanghai, China. The PCR experiment used 50 μL reaction volume with 200 μM deoxynucleoside triphosphates and 0.4 μM forward or reverse primers, and 2 U of Taq DNA polymerase from TaKaRa, Japan. The experimental protocol involved the utilization of specific cycling parameters for the cycling process. This comprised a total of 35 cycles, with each cycle consisting of denaturation at a temperature of 94 °C for 45 seconds, followed by annealing at 55 °C for 45 seconds and extension at 72 °C for 45 seconds. Additionally, a final extension step was performed at 72 °C for 10 minutes. Negative controls for PCR were conducted to assess the presence of contamination without any template of DNA. Negative PCR controls were removed from sequencing because they had no PCR result [35]. Reaction mixtures per sample were mixed in triplicate and purified using an agarose gel DNA purification kit (TaKaRa). Before sequencing, PCR products were mixed in equimolar amounts (10 pg each sample).

Reference:

[32] Hebert, P.D.N.; Stoeckle, M.Y.; Zemlak, T.S.; Francis, C.M. Identification of birds through DNA barcodes. *PLOS Biol.* **2004**, *2*, e312. DOI: 10.1371/journal.pbio.0020312.

[33] Xiang, X.; Zhang, F.; Fu, R.; Yan, S.; Zhou, L. Significant differences in bacterial and potentially pathogenic communities between sympatric hooded crane and greater white-fronted goose. *Front. Microbiol.* **2019**, *10,* 163. DOI: 10.3389/fmicb.2019.00163.

[34] Biddle, J.F.; Fitz-Gibbon, S.; Schuster, S.C.; Brenchley, J.E.; House, C.H. Metagenomic signatures of the Peru Margin subseafloor biosphere show a genetically distinct environment. *Proc. Natl. Acad. Sci. U.S.A.* **2008**, *105*, 10583-10588. DOI: 10.1073/pnas.0709942105.

[35] Salter, S.J.; Cox, M.J.; Turek, E.M.; Calus, S.T.; Cookson, W.O.; Moffatt, M.F.; Walker, A.W. Reagent and laboratory contamination can critically impact sequence-based microbiome analyses. *BMC Biol.* **2014**, *12*, 1-12. DOI: 10.1186/s12915-014-0087-z.

Table S1: The different diets of three hosts.

Hosts	Diets
**Bean goose**	Gramineous plants [26], *Alopecurus aequalis* and Water Chestnut Trapa maximomczii fruits [27], spilt rice and winter wheat [28], dicotyledons and moss [29]
**Hooded crane**	Spilt rice and winter wheat [28], *Potamogeton malaianus*, *Vallisneria natans* [20], underground corms of *Scirpus mariqutter*, cereal grains and gastropods [30]
**Domestic goose**	Artificial throw food (i.e., paddy) [31], grass and aquatic plant

Reference:

[26] Zhao, K.; Zhou, D.; Ge, M.; Zhang, Y.; Li, W.; Han, Y.; Shi, S. Intestinal Microbiota of *Anser fabalis* Wintering in Two Lakes in the Middle and Lower Yangtze River Floodplain. *Animals* **2023**, *13*, 707. DOI: 10.3390/ani13040707.

[27] Zhao, M.; Cao, L.; Fox, A.D. Distribution and diet of wintering Tundra Bean Geese *Anser fabalis* serrirostris at Shengjin Lake. Yangtze River floodplain, China. *Wildfowl* **2010**, *60*, 52-63.

[28] Lei, C.A.O.; Barter, M.; Meijuan, Z.H.A.O.; Haoxian, M.E.N.G.; Zhang, Y.A. systematic scheme for monitoring waterbird populations at Shengjin Lake, China: methodology and preliminary results. *Avian Res.* **2011**, *2*, 1-17. DOI: 10.5122/cbirds.2011.0001.

[29] Zhang, P.; Yeai, Zou.; Xie, Y.; Zhang, S.; Chen, X.; Li, F. Hydrology-driven responses of herbivorous geese in relation to changes in food quantity and quality. *Ecol. Evol.* **2020**, *10*, 5281-5292. DOI: 10.1002/ece3.6272.

[20] Zheng, M.; Zhou, L.; Zhao, N.; Xu, W. Effects of variation in food resources on foraging habitat use by wintering Hooded Cranes (*Grus monacha*). *Avian Res.* **2015**, *6*, 1-10. DOI: 10.1186/s40657-015-0020-3.

[30] Cui, Y.; Tang, Y.; Yang, S.; Wu, W.; Feng, X.; Ma, Q.; Ma, Z. Changes in wintering Hooded Cranes and their habitats at Chongming Dongtan over the past 20 years. *Avian Res.* **2023**, *14*, 100083. DOI: 10.1016/j.avrs.2023.100083.

[31] Xiang, X.; Jin, L.; Yang, Z.; Zhang, N.; Zhang, F. Dramatic shifts in intestinal fungal community between wintering Hooded Crane and Domestic Goose. *Avian Res.* **2021**, *12*, 1-6. DOI: 10.1186/s40657-020-00238-1.

Table S2: The distribution of data was examined by Shapiro-Wilk's test (Normal distribution: $P > 0.05$; n = 20 for BG, HC and DG, respectively). The data of non-normal distribution were transformed by square root transformation. BG: Bean Goose; HC: Hooded Crane; DG: Domestic Goose.

	**Shapiro-Wilk's test (*P*-value)**			**Distribution**
	**BG**	**HC**	**DG**	
Bacterial ASV richness	0.785	0.301	0.874	Normal
Bacterial phylogenetic diversity	0.920	0.298	0.716	Normal
Bacterial βNTI value	0.125	0.142	0.170	Normal
Pathogenic diversity (square root transformation)	0.188	0.067	0.137	Normal
Pathogenic relative abundance (square root transformation)	0.113	0.105	0.109	Normal
Firmicutes relative abundance	0.228	0.081	0.123	Normal
Proteobacteria relative abundance (square root transformation)	0.130	0.421	0.055	Normal
Actinobacteria relative abundance (square root transformation)	0.607	0.815	0.266	Normal
Bacteroidetes relative abundance (square root transformation)	0.060	0.128	0.231	Normal
Lactobacillus relative abundance	0.078	0.158	0.062	Normal
Pseudomonas relative abundance (square root transformation)	0.125	0.241	0.117	Normal
Enterococcus relative abundance (square root transformation)	0.139	0.088	0.353	Normal
Bacillus relative abundance (square root transformation)	0.692	0.700	0.559	Normal

Table S3: The potential pathogens were detected in guts of bean goose, hooded crane and domestic goose. BG: Bean Goose; HC: Hooded Crane; DG: Domestic Goose.

**Pathogens**	**Symptom**	**Hosts**
Nocardia farcinica	Brain abscess	Human, pig, livestock [45-48]
Streptococcus suis	Meningitis	Human, pig [49]
Roseomonas ludipueritiae	Skin infection	Human, sheep [50,51]
Campylobacter canadensis	Cellulitis, sepsis	Human, bird [52,53]
Campylobacter jejuni	Diarrhea, fever, abdominal cramps	Human, bird, poultry [54,55]
Burkholderiales bacterium	Bacterial infection	Human [56]
Nocardia nova	Abscesses	Human, animals [57]
Clostridium botulinum	Botulism	Human, cows [58,59]
Anaerobiospirillum succiniciproducens	Sepsis, bacteremia and diarrhea	Human, cat and dog [60-62]
Clostridium perfringens	Gas gangrene, food poisoning, enterotoxemia	Human, animals, poultry [63,64]

Reference:

[45] Boamah, H.; Puranam, P.; Sandre, R.M. Disseminated *Nocardia farcinica* in an immunocompetent patient. *IDCases* **2016**, *6*, 9-12. DOI: 10.1016/j.idcr.2016.08.003.

[46] Wallace, R.J.; Tsukamura, M.; Brown, B.A.; Brown, J.; Steingrube, V.A.; Zhang, Y.S.; Nash, D.R. Cefotaxime-resistant *Nocardia asteroides* strains are isolates of the controversial species *Nocardia farcinica. J. Clin. Microbiol.* **1990**, *28*, 2726-2732. DOI: 10.1128/jcm.28.12.2726-2732.1990.

[47] Zhu, W., Shu, Z., Song, H.A rare case of a hard-to-heal ulcer caused by pulmonary *Nocardia* infection. *Journal of Wound Care.* **2024**, *33*, 197-200. DOI: 10.12968/jowc.2024.33.3.197.

[48] Faccin, M.; Wiener, D.J.; Rech, R.R.; Santoro, D.; Rodrigues Hoffmann, A. Common superficial and deep cutaneous bacterial infections in domestic animals: A review. *Vet. Pathol.* **2023**, *60*, 796-811. DOI: 10.1177/03009858231176558.

[49] Hughes, J.M.; Wilson, M.E.; Wertheim, H.F.L.; Nghia, H.D.T.; Taylor, W.; Schultsz, C. *Streptococcus suis*: an emerging human pathogen. *Clin. Infect. Dis.* **2009**, *48*, 617-625. DOI: 10.1086/596763.

[50] Tian, Z.; Lu, S.; Jin, D.; Yang, J.; Pu, J.; Lai, X.H.; Xu, J. *Roseomonas wenyumeiae* sp. nov., isolated from faeces of Tibetan antelopes (*Pantholops hodgsonii*) on the Qinghai–Tibet Plateau. International Journal of *Systematic and Evolutionary Microbiology* **2019**, *69*, 2979-2986. DOI: 10.1099/ijsem.0.003479.

[51] Romano-Bertrand, S.; Bourdier, A.; Aujoulat, F.; Michon, A.L.; Masnou, A.; Parer, S.; Jumas-Bilak, E. Skin microbiota is the main reservoir of *Roseomonas mucosa*, an emerging opportunistic pathogen so far assumed to be environmental. *Clin. Microbiol. Infect.* **2016**, *22*, 737. e1-737. e7. DOI: 10.1016/j.cmi.2016.05.024.

[52] Marinella, M.A. Cellulitis and sepsis due to *Sphingobacterium*. *Jama* **2002**, *288*, 1985-1985. DOI: 10.1001/jama.288.16.1985-a.

[53] Waldenström, J.; On, S.L.; Ottvall, R.; Hasselquist, D.; Harrington, C.S.; Olsen, B. Avian reservoirs and zoonotic potential of the emerging human pathogen *Helicobacter canadensis*. *Appl. Environ. Microb.* **2003**, *69*, 7523-7526. DOI: 10.1128/aem.69.12.7523-7526.2003.

[54] Robino, P.; Tomassone, L.; Tramuta, C.; Rodo, M.; Giammarino, M.; Vaschetti, G.; Nebbia, P. Prevalence of *Campylobacter jejuni*, *Campylobacter coli* and enteric *Helicobacter* in

domestic and free living birds in North-Western Italy. *Schweizer Archiv fur Tierheilkunde***. 2010**, *152*, 425. DOI: 10.1024/0036-7281/a000094.

[55] Acheson, D.; Allos, B.M. *Campylobacter jejuni* infections: update on emerging issues and trends. *Clin. Infect. Dis.* **2001**, *32*, 1201-1206. DOI: 10.1086/319760.

[56] LiPuma, J.J. Update on the *Burkholderia cepacia* complex. Curr. *Opin. Pulm. Med.* **2005**, *11*, 528-533. DOI: 10.1097/01.mcp.0000181475.85187.ed.

[57] Wilson, J.W. Nocardiosis: updates and clinical overview. *Mayo Clin. Proc.* **2012**, *87*, 403-407. DOI: 10.1016/j.mayocp.2011.11.016.

[58] Barash, J.R.; Arnon, S.S. A novel strain of *Clostridium botulinum* that produces type B and type H botulinum toxins. *J. Infect. Dis.* 2014, *209*, 183-191. DOI: 10.1093/infdis/jit449.

[59] Krüger, M.; Shehata, A.A.; Grosse-Herrenthey, A. Relationship between gastrointestinal dysbiosis and *Clostridium botulinum* in dairy cows. *Anaerobe* **2014**, *27*, 100-105. DOI: 10.1016/j.anaerobe.2014.03.013.

[60] Koshy, J.; Aronson, J.F.; Vishwanath, B.; Williams-Bouyer, N. *Anaerobiospirillum succiniciproducens* sepsis in an autopsy patient: A troublesome diagnostic workup. *IDCases* **2014**, *1*, 50-52. DOI: 10.1016/j.idcr.2014.06.004.

[61] Youssef, G.V.; Kar, P.; Mitra, S. *Anaerobiospirillum succiniciproducens* prosthetic joint infection. *Anaerobe* **2022**, *79*, 102689. DOI: 10.1016/j.anaerobe.2022.102689.

[62] Schaumburg, F.; Dieckmann, R.; Schmidt-Bräkling, T.; Becker, K.; Idelevich, E.A. First description of an *Anaerobiospirillum succiniciproducens* prosthetic joint infection. *New Microbes and New Infections* **2017**, *18*, 1-2. DOI: 10.1016/j.nmni.2017.03.001.

[63] Rood J.I. Virulence genes of *Clostridium perfringens*. *Annu. Rev. Microbiol.* **1998**, *52*, 333-360. DOI: 10.1146/annurev.micro.52.1.333.

[64] Immerseel, F.V.; Buck, J.D.; Pasmans, F.; Huyghebaert, G.; Haesebrouck, F.; Ducatelle, R. *Clostridium perfringens* in poultry: an emerging threat for animal and public health. *Avian Pathol.* **2004**, *33*, 537-549. DOI: 10.1080/03079450400013162.

Table S4: Co-occurrence network topological features. BG: Bean Goose; HC: Hooded Crane; DG: Domestic Goose.

Treatment	BG	HC	DG
Nodes	352	349	369
Edges	15056	13468	8494
Density	0.122	0.111	0.063
Modularity	0.575	0.677	0.680
Degree (Average)	42.77	38.59	23.02
Network diameter	10	8	12
Clustering coefficient	0.78	0.75	0.69
Path length (Average)	2.965	3.131	3.847
Closeness centrality (Average)	0.0098	0.0095	0.0014
Betweenness centrality (Average)	340.9	518.2	370.7
Eigenvector centrality (Average)	0.206	0.140	0.224

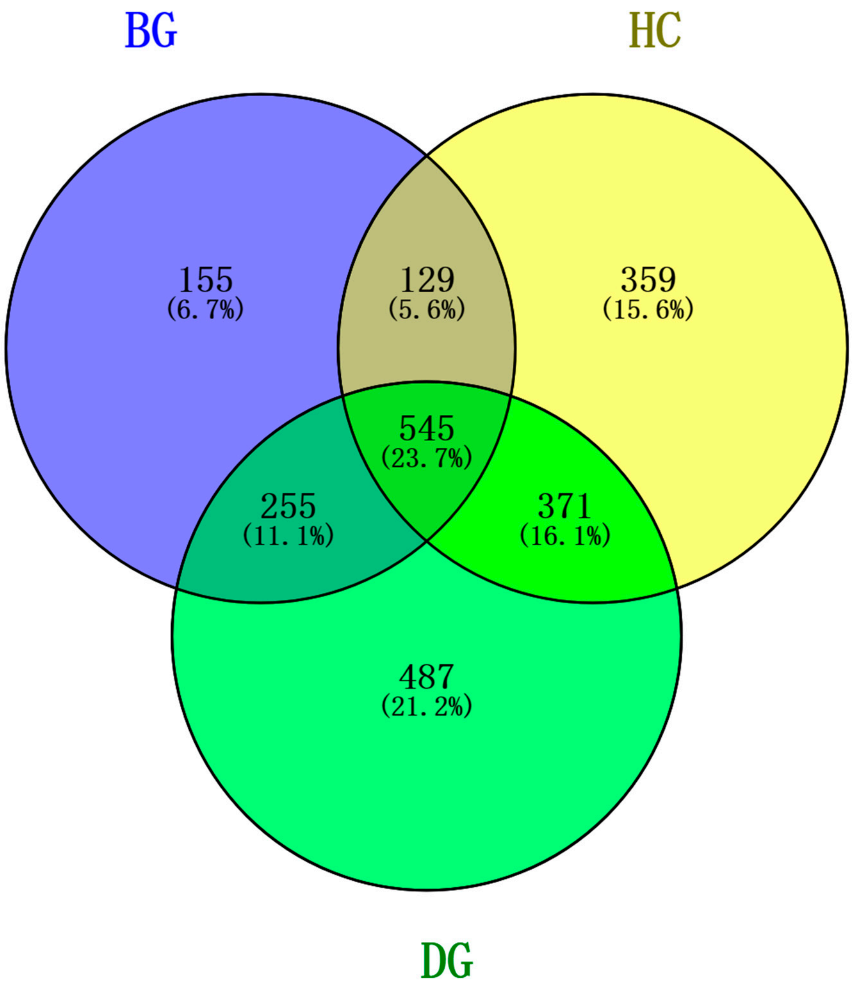

Figure S1: The Venn diagram showing the unique and shared gut bacterial ASVs in guts of bean goose, hooded crane and domestic goose. BG: Bean Goose; HC: Hooded Crane; DG: Domestic Goose. ASV: Amplicon Sequence Variation.

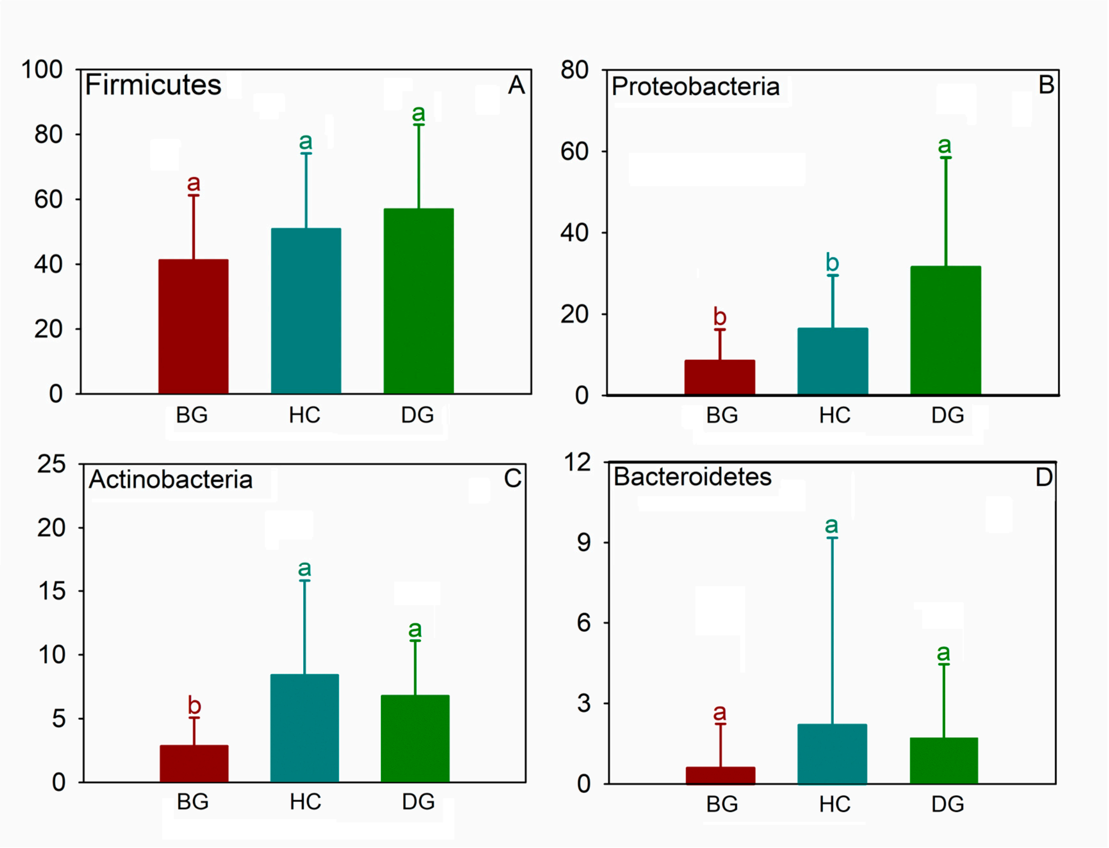

Figure S2: Relative abundance of the dominant bacterial phyla. Letters represent significant differences from the One-way ANOVA ($P < 0.05$; n = 20 for BG, HC and DG, respectively). BG: Bean Goose; HC: Hooded Crane; DG: Domestic Goose.

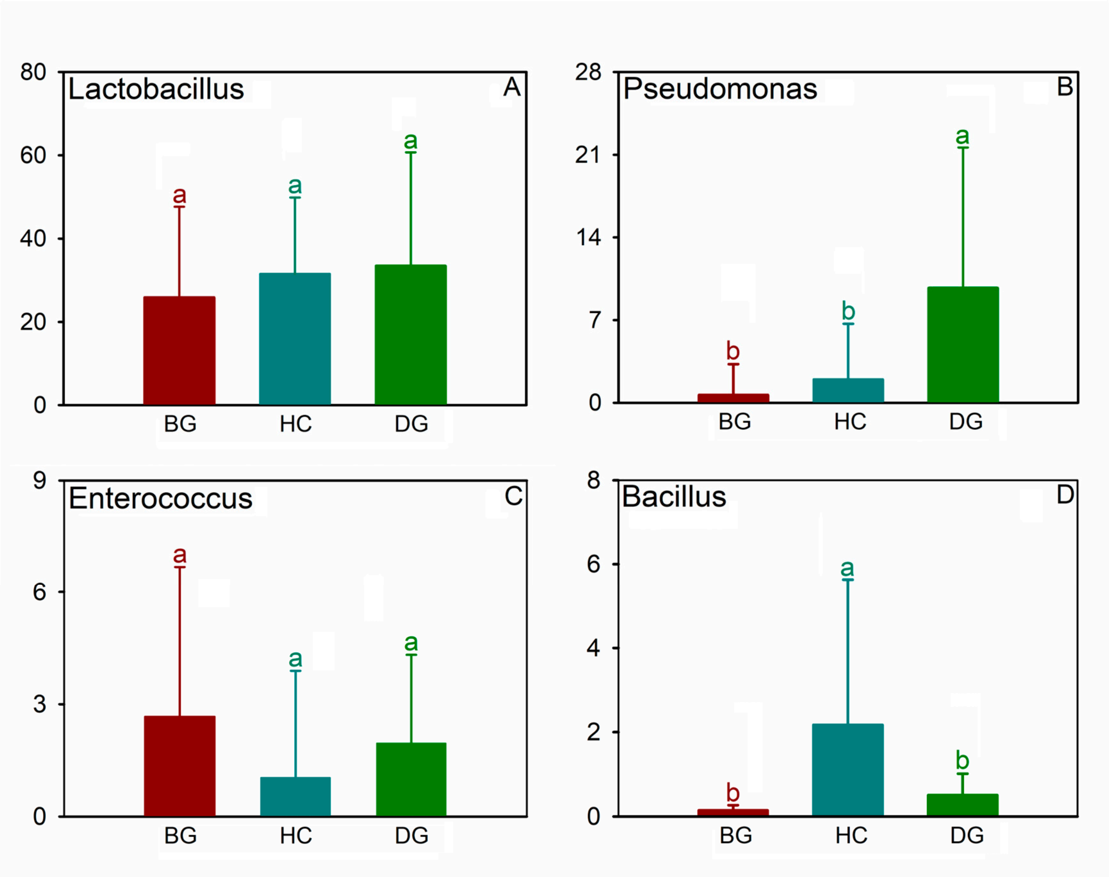

Figure S3: Relative abundance of the dominant bacterial genus. Letters represent significant differences from the One-way ANOVA ($P < 0.05$; n = 20 for BG, HC and DG, respectively). BG: Bean Goose; HC: Hooded Crane; DG: Domestic Goose.

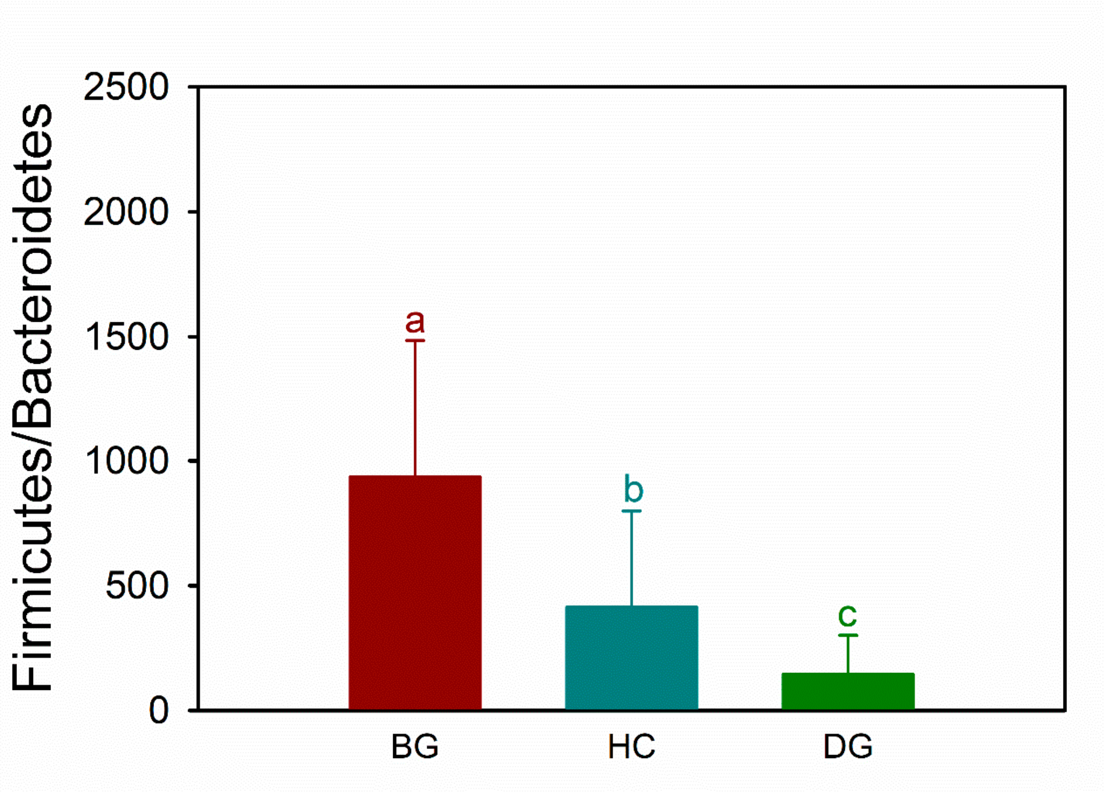

Figure S4: The ratio of relative abundance of Firmicutes and Bacteroidetes. Letters represent significant differences from the One-way ANOVA ($P < 0.05$; n = 20 for BG, HC and DG, respectively). BG: Bean Goose; HC: Hooded Crane; DG: Domestic Goose.

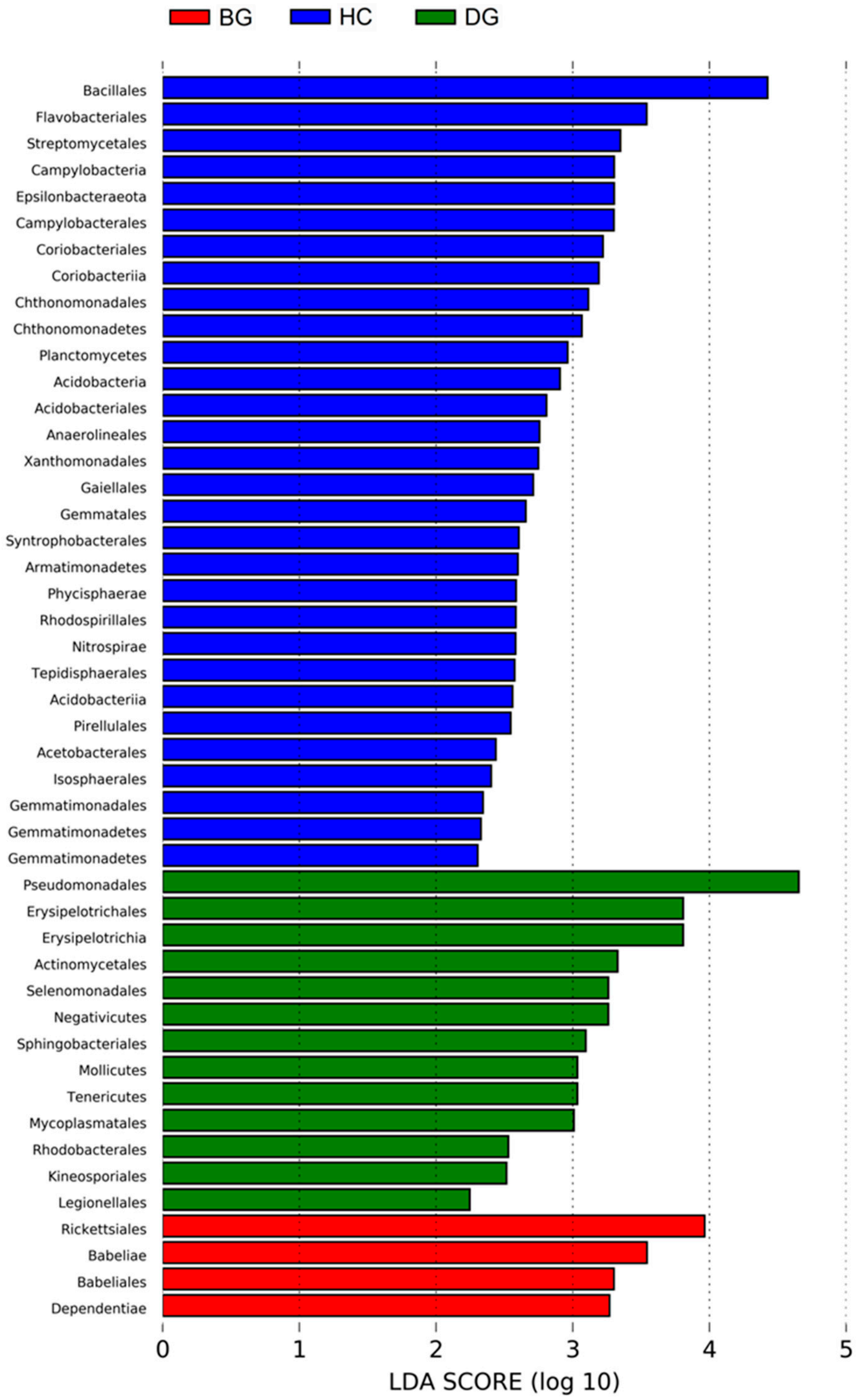

Figure S5: Identified phylotype biomarkers ranked by effect size with the alpha value was < 0.05 in different hosts. The phylotype biomarkers were identified as significantly abundant when samples in bean goose, hooded crane and domestic goose were compared (n = 20 for BG, HC and DG, respectively). BG: Bean Goose; HC: Hooded Crane; DG: Domestic Goose.

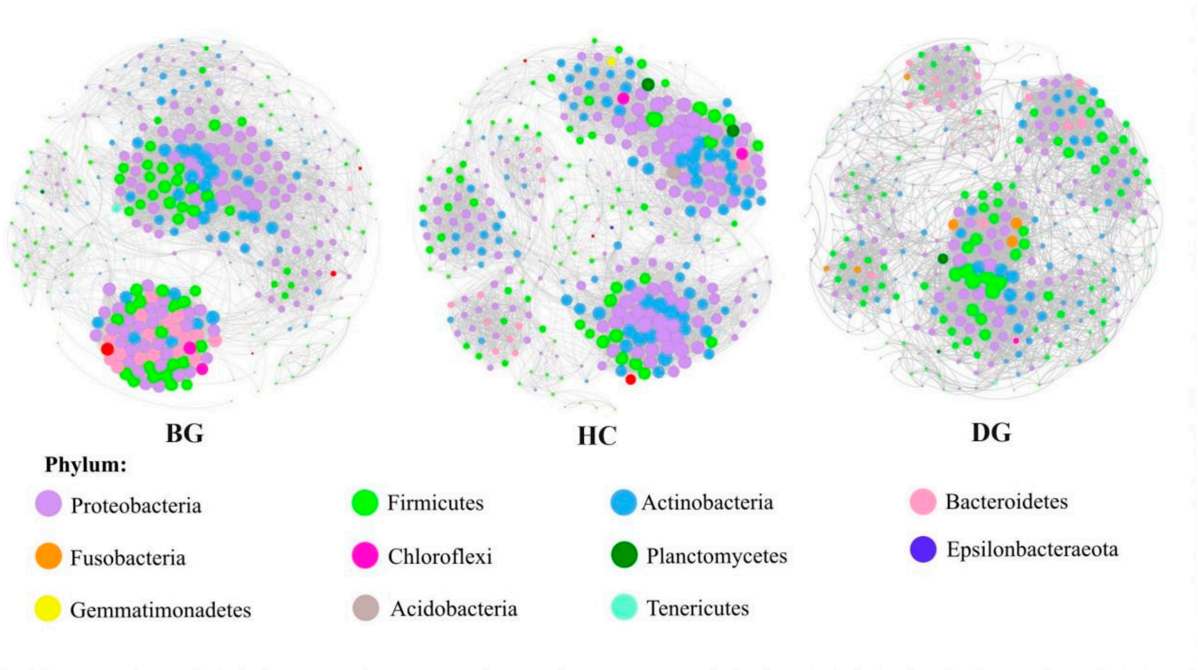

Figure S6: The co-occurrence network structure of gut bacterial community for bean goose, hooded crane and domestic goose. with ASV's relative abundance > 0.01% at phylum level among three stages. N = 20 for BG, HC and DG, respectively. BG: Bean Goose; HC: Hooded Crane; DG: Domestic Goose.

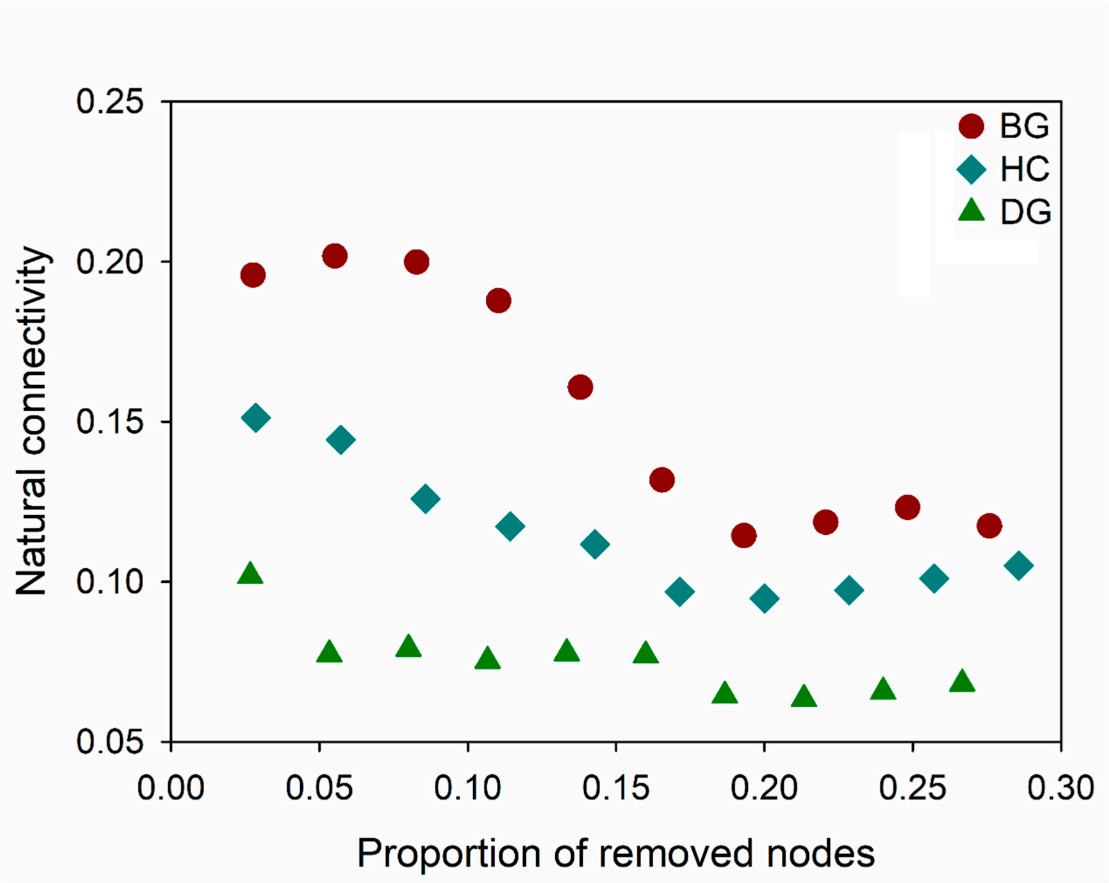

Figure S7: The stability of co-occurrence network. BG: Bean Goose; HC: Hooded Crane; DG: Domestic Goose.
